# Imputation models and error analysis for phase contrast MR cerebral blood flow measurements

**DOI:** 10.3389/fphys.2023.1096297

**Published:** 2023-02-20

**Authors:** Payal Shah, Eamon Doyle, John C. Wood, Matthew T. Borzage

**Affiliations:** ^1^ Division of Cardiology, Department of Pediatrics, Children’s Hospital Los Angeles, Los Angeles, CA, United States; ^2^ Department of Radiology, Children’s Hospital Los Angeles, Los Angeles, CA, United States; ^3^ Division of Neonatology, Department of Pediatrics, Children’s Hospital Los Angeles, Keck School of Medicine, Fetal and Neonatal Institute, University of Southern California, Los Angeles, CA, United States

**Keywords:** magnetic resonance imaging (MRI), cerebral blood flow (CBF), phase contrast (PC), internal carotid artery (ICA), vertebral artery

## Abstract

Cerebral blood flow (CBF) supports brain metabolism. Diseases impair CBF, and pharmacological agents modulate CBF. Many techniques measure CBF, but phase contrast (PC) MR imaging through the four arteries supplying the brain is rapid and robust. However, technician error, patient motion, or tortuous vessels degrade quality of the measurements of the internal carotid (ICA) or vertebral (VA) arteries. We hypothesized that total CBF could be imputed from measurements in subsets of these 4 feeding vessels without excessive penalties in accuracy. We analyzed PC MR imaging from 129 patients, artificially excluded 1 or more vessels to simulate degraded imaging quality, and developed models of imputation for the missing data. Our models performed well when at least one ICA was measured, and resulted in *R* ^2^ values of 0.998–0.990, normalized root mean squared error values of 0.044–0.105, and intra-class correlation coefficient of 0.982–0.935. Thus, these models were comparable or superior to the test-retest variability in CBF measured by PC MR imaging. Our imputation models allow retrospective correction for corrupted blood vessel measurements when measuring CBF and guide prospective CBF acquisitions.

## Introduction

The brain requires a significant fraction of the metabolic support of the body, and it lacks the capacity to buffer disruptions in blood supply. Thus, adequate cerebral blood flow (CBF) is vital to brain health (Lassen, 1959) and CBF measurements span many areas of brain research. CBF varies markedly with brain development throughout infancy, childhood, and senescence. (Oshima et al., 2002; Liu et al., 2019; Paniukov et al., 2020). Altered CBF is associated with cardiovascular risk, (Jennings et al., 2013; King et al., 2018), small vessel disease, (Yu et al., 2020), Alzheimer’s dementia, (Leijenaar et al., 2017), white matter lesions, (Hanaoka et al., 2016), anemia, (Borzage et al., 2016), stroke risk in sickle cell disease, (Vernooij et al., 2008; Prohovnik et al., 2009), and higher risk of non-cardiovascular mortality in the elderly. (Sabayan et al., 2013). CBF is a predictive biomarker for patients with severe depression, (Leaver et al., 2019), and CBF is modulated by pharmacological agents including substances of abuse, (Chen et al., 2016), alcohol, (Christie et al., 2008), and anesthetics. (Oshima et al., 2002).

The gold standard for CBF measurements are Kety-Schmidt tracer methods for whole brain measurements and Positron Emitting Tomography (PET) for spatially resolved measurements. However, both are invasive, making them poor candidates for neurovascular screening or research applications. A non-invasive alternative CBF measurement is arterial spin labeling (ASL) magnetic resonance imaging (MRI), which uses magnetically labeled water protons in the blood as endogenous tracers to estimate brain perfusion. However, quantitation of ASL is limited by the measurement or assumption of multiple parameters, including blood-brain partition coefficient, T1 of blood, T1 of brain tissue, labeling efficiency, and arterial transit time. (Alsop et al., 2010). Estimates of each of these key parameters are provided in the literature, however experimental methods demonstrate variability for each: the blood-brain partition coefficient varies with age, underlying pathology and brain region; (Thalman et al., 2019) T1 of blood varies with red cell characteristics and hematocrit; (Lu et al., 2004) labeling efficiency is a confounding variable that depends on the blood velocity and labelling schema; (Robertson et al., 2017) and arterial transit time varies with the health, age (MacIntosh et al., 2015; Dai et al., 2017) and brain region. (Thomas et al., 2006). The reproducibility of ASL is moderate (ICC 0.74–0.78); (Yang et al., 2019) and ASL acquisitions are lengthy, with typical scan times of 5–7 min. As a result, ASL is excellent at determining relative brain perfusion in specific regions, but less suitable for measuring absolute blood flow.

Alternatively, phase contrast (PC) MRI of the internal carotid (ICA) or vertebral (VA) arteries provides rapid and robust quantification of global CBF (ml/min). The measurement yielded by PC MRI can be normalized to brain volume from brain mass estimates derived from anatomic imaging to estimate global brain perfusion in ml/100 g/min. PC MRI is reproduceable between users (ICC 0.97–0.99) and has a low coefficient of variation (4%–9%) for serial measurements. (Koerte et al., 2013; Liu et al., 2014; Sakhare et al., 2019). If phasic variation is unimportant, then accurate CBF can be collected without cardiac gating thereby greatly accelerating the acquisition. Most importantly, PC-MRI does not require modeling or parameter estimation, making it robust across pathological states.

One barrier to deploying PC MRI in clinical practice is the need to minimize contributions from partial-voluming or off-axis flow. The MR operator prevents these issues by carefully localizing the imaging plane orthonormal to the vessel being measured. The four head vessels are not parallel, thus optimal measurement of one of vessel compromises measurements from the others. To overcome this problem, the MR operator can use four PC MR images at the cost of a four-fold increase in time. However, manual optimization of each image remains dependent on operator skill. Automated methods for analyzing the anatomy of patients and planning ideal locations of four PC MR imaging slices have been proposed, but not implemented by MR scanner vendors. (Liu et al., 2014). Alternatively, placing a “best-guess” single PC MR imaging slice to measure CBF is methodologically simple and fast, but is inherently suboptimal and occasionally results in one or more vessels being too oblique for acceptable quantitation.

We postulated that flows in each vessel were sufficiently correlated with one another so that imputation could compensate for measurements corrupted by motion or obliquity, provided that one or more vessels remain measurable. To test this hypothesis, we calculated the internal correlations among the head vessels, developed a table of mathematical models to impute the flow from corrupted arterial flow measurements, and assessed the resulting error introduced by our imputations.

## Materials and methods

### Patient demographics

This study is a secondary analysis of existing data, which was originally approved by the Children’s Hospital Los Angeles Committee on Clinical Investigations (CCI 11–00083). Informed consent was obtained from N = 129 patients recruited between 2012 and 2017. This cohort included patients with sickle cell disease (N = 55), healthy control patients (N = 42), and patients suffering from various hemoglobinopathies (N = 32). These patients were 23.5 ± 9.7 years (range 9–61 years) old (59M, 70F). Their hematocrits were 32.9% ± 7.2% and 33% (N = 42) were on chronic transfusion. (Borzage et al., 2016).

### Image acquisition

**FIGURE 1:**
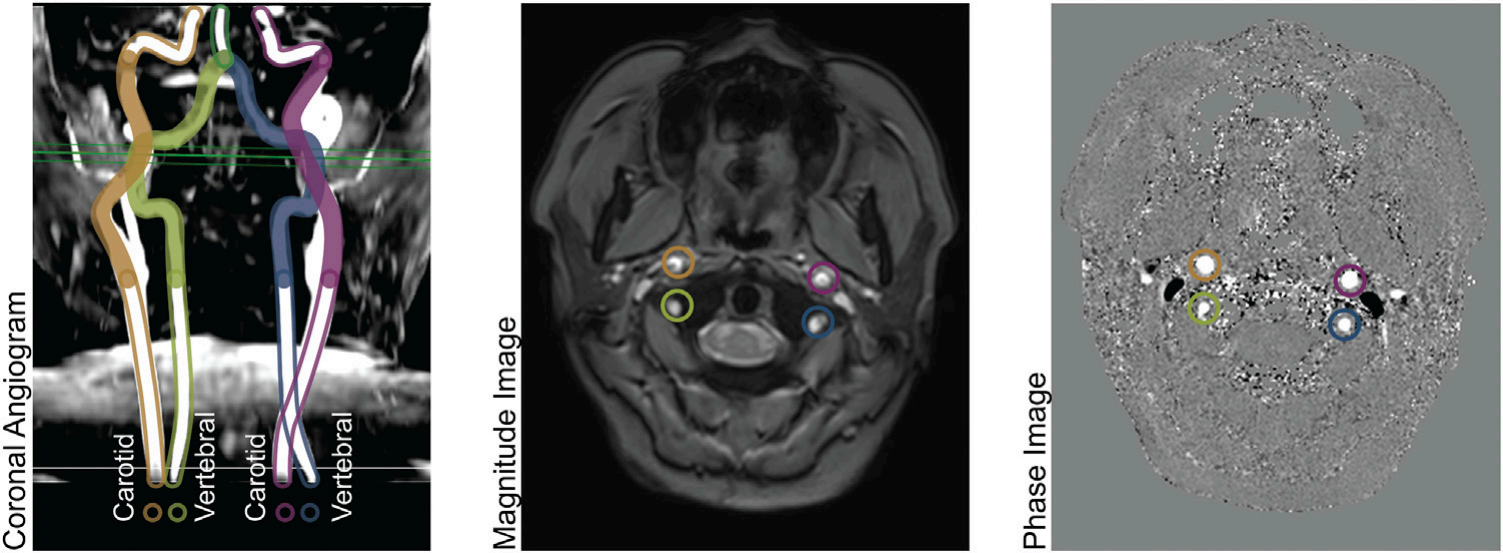
Left panel: Coronal Angiogram showing the carotid and vertebral arteries. The solid-colored portion of the vessels are above the bifurcation of the common carotid arteries, and below the level of the basilar artery. The green horizontal plane shows the location of the phase contrast slice. Center panel: a magnitude image; and right panel: a phase image, both acquired at the level indicated in the left panel.

The imaging methods are reported elsewhere in detail and summarized here. (Borzage et al., 2016). We obtained all images with a 3T Philips Achieva and eight-element head coil. A magnetic resonance angiogram localized the vessels in the neck, and a PC MR imaging plane was placed approximately 1 cm above the carotid bifurcation. The angiogram was collected in the axial plane with inline reformatting into sagittal and coronal planes to facilitate orthogonal placement of the PC imaging plane. Image parameters for the PC MR examination were as follows: repetition time, 12.3 ms; echo time, 7.5 ms; field of view, 260 mm; thickness, 5 mm; signal averages, 10; acquisition matrix, 204 × 201; reconstruction matrix, 448 × 448; bandwidth, 244 Hz/pixel; and velocity encoding gradient, 200 cm/s. For this retrospective data analysis, we retained only one PC MR image per patient. Figure 1 demonstrates the coronal angiogram showing the carotid and vertebral arteries together with the magnitude and phase images.

### Image processing

We performed all phase-contrast image analysis using MATLAB (The MathWorks, Natick, MA). We thresholded the complex difference image to identify moving voxels (defined as greater than the mean plus two standard deviations of stationary voxels sampled from a non-vascular region). We fit the phase differences of stationary voxels using a two-dimensional second-order polynomial to remove the background phase. We identified vessel boundaries using a Canny edge-detector of the complex difference image, dilating the edge by a single voxel, and excluding any stationary voxels. We calculated the blood flow in each artery by summing the blood velocities (cm/s) within the vessel multiplied by the voxel area (cm^2^). When the automatic edge detection failed (<5% of the time), the carotid or vertebral artery boundaries were identified manually by an MR researcher (JCW) with 22 years of experience analyzing PC MR images.

### Modeling cerebral blood flow

We identified all 16 possible scenarios wherein combinations of two, one, or zero ICAs or VAs might be able to be analyzed from an image (Table 1). We synthesized each of these 16 scenarios from each PC MR image to simulate the effects of a sub-optimal image which failed to assess the four vessels to be analyzed. We applied a standard least-squares model to model the total cerebral blood flow (CBF) as a function of the vessels able to be analyzed. In scenarios where two ICAs or two VAs were analyzed, we simplified and reduced the degrees of freedom in the models by calculating the total anterior (sum of ICAs) or posterior (sum of VAs) flow. We evaluated the quality of each model using root mean squared error, intra-class correlation coefficients (ICC), *R* ^2^ statistic. The ICC (Lassen, 1959; Liu et al., 2019) is a two-way random, single measures absolute agreement between model 0 (gold standard) and models 1-8, calculated using MATLAB. We also performed Bland-Altman analyses of all imputed models compared to total CBF. We calculated the biases as the mean difference between model 0 and each other model, and the 95% limits of agreement as twice the standard deviation of the differences of individual measurements between model 0 and each other model.

**TABLE 1:** Models for computing cerebral blood flow.

Number of usable vessels		Model	Cerebral blood flow equation	RMSE	Normalized RMSE	R-Squared	ICC(2,1)
ICAs	VAs						
2	2	0	CBF = Anterior + Posterior	00.00	0.000	1.000	1.000
	1	1	CBF = 1.226 × Anterior + 0.933 × VA	41.00	0.044	0.998	0.982
	0	2	CBF = 1.426 × Anterior	69.89	0.075	0.995	0.973
1	2	3	CBF = 1.866 × ICA + 1.145 × Posterior	57.19	0.061	0.997	0.983
	1	4	CBF = 2.419 × ICA + 0.983 × VA	78.75	0.084	0.994	0.948
	0	5	CBF = 2.841 × ICA	98.20	0.105	0.990	0.935
0	2	6	CBF = 3.219 × Posterior	159.46	0.171	0.974	0.877
	1	7	CBF = 5.816 × VA	323.45	0.346	0.885	0.662
	0	8	CBF = 933.656	297.88	0.319	NA	0.000

Models for computing cerebral blood flow when vessels are missing. The models are independent of the lateral location of the vessel(s) that are imputed thereby allowing us to omit models for the combinations of different left *versus* right vessels (See Figure 3, Results). Models 1-7 are computed with an intercept of zero; model eight is the mean CBF in this study. The models followed the anticipated pattern wherein missing ICAs *versus* VAs contributed more error (e.g. model 3 *versus* 1). Abbreviations: cerebral blood flow (CBF), internal carotid artery (ICA), vertebral artery (VA), root mean square error (RMSE), intra-class correlation coefficient (ICC). An online calculator for these models is provided: https://brainflow.science/impute-cbf.

## Results

We report the total cerebral blood flow (933.7 ± 297.9), anterior circulation (sum of ICAs, 652.6 ± 209.2), and posterior circulation (sum of VAs, 281.0 ± 106.2). We also report the flow in the individual arteries: left ICA (328.1 ± 110.5), right ICA (324.9 ± 105.7), left VA (145.9 ± 76.8), and right VA (134.9 ± 53.1), all values are reported in units of ml/min as mean ± standard deviation. (Figure 1, Left). We also report the ratio of flow in individual arteries *versus* total cerebral blood flow, which are: left ICA (0.352 ± 0.039), right ICA (0.348 ± 0.039), left VA (0.154 ± 0.049), and right VA (0.147 ± 0.044), all values are unitless, and reported as mean ± standard deviation. (Figure 1, Right).

We evaluated all four ratios (left ICA-CBF, right ICA-CBF, left VA-CBF, right VA-CBF) with an ANOVA, and tested all six pairwise comparisons with Tukey-Kramer HSD. Only two pairs of ratios were not different: the left ICA-CBF *versus* right ICA-CBF ratios (*p* = 0.549), and the left VA-CBF *versus* right VA-CBF ratios (*p* = 0.549). Thus, we simplified from 16 models to eight using symmetry of the left and right CBF ratios. All other pairs of ratios were different (*p* < 0.0001).

We created eight statistical models of CBF using standard least squares simple linear regression. We computed the performance statistics for the models, including R^2^value, root mean square error (RMSE), normalized RMSE and ICC to evaluate the performance and the reliability of the models (Table 1). These models are numbered from the case with all arteries present (model 0), in ascending order of increasing expected error to the model with the most error because there are no arteries present (model 8). As anticipated from model 1 to model 8, the error in imputation (RMSE) increased and the *R* ^2^ decreased. All these models were statistically significant (*p* < 0.0001), except when there were no usable blood vessels (model 8).

**FIGURE 2:**
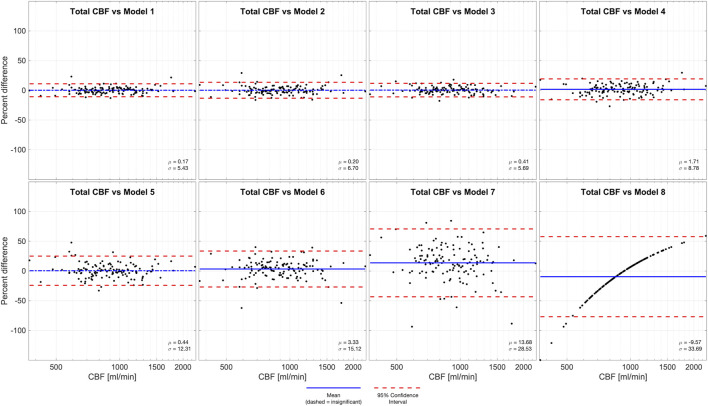
Bland-Altman analysis of total CBF quantified by PC in four vessels (model 0) *versus* various models of one or more missing vessels. Blue line demonstrates bias, dashed blue line demonstrates insignificant (*p* > 0.05) bias, and red dash lines demonstrate 95% confidence intervals. These results suggest that it is important to successfully capture at least one ICA or both VAs to impute CBF measurements.

We performed Bland-Altman analyses of models 1-8 compared to total CBF (model 0), demonstrated in Figure 2. Models 1-3 and 5 are all statistically unbiased (*p* > 0.05), models 4 and 6 had small biases (1.71% and 3.33%, respectively). Models 1-4 had narrow limits of agreement, with standard deviations of 5.43%–8.78%. Models 5-6 and had wider limits of agreement, with standard deviations of 12.31%–15.12%. Model 7, which was derived from a measurement in one VA, and it performed exceptionally poorly (Bias −9.57%, standard deviation 28.53%). Thus, model 7 had similar performance to model 8 (Bias −9.57% standard deviation 33.69%) wherein flow is assumed equal to the population mean.

## Discussion

Our models were suitable to impute CBF for all scenarios wherein at least one ICA was usable (Model 1–5). We do not recommend attempting to impute CBF without any ICA measurements (models 6–8) because of their poor performance. We included models 6 and 7 because they were valid correlates of total cerebral blood flow, and model 8 (mean CBF) for completeness. We recommend the reader interpret our model performances in context of the accuracy and precision needed for each specific use case for measuring CBF. In any situation in which these models are used they add error *versus* the ideal situation wherein all four vessels are perfectly measured in one PC MR image. Thus, if any of the models are used, the error they introduce should be considered and the modeled results should be held with appropriate uncertainty.

The best comparisons of our model performance would be *versus* the performance of other CBF imputation models that use PC MR data. However, because our motivation for these models was the lack of any extant model we cannot ascertain how our models perform *versus* other approaches. As an imperfect alternative, we provide the reader with examples of alternative studies of CBF correlations using different measurement and validation approaches as context for our model performance. The correlation of arterial spin labeling *versus* ^15^O positron emission tomography (*R* ^2^ 0.47 total CBF and *R* ^2^ 0.48 voxelwise blood flow); (Heijtel et al., 2014) and the intraclass correlation of test-retest measurements with PC MR with no repositioning of slice or patient (ICC 0.79). (Sakhare et al., 2019). provide context to judge our imputation performance *versus* other methods and errors commonly encountered by those who measure CBF.

Imputation error increased more when an ICA was corrupted than a VA. This was anticipated because the ICA makes a greater contribution to CBF than a VA. Thus, a single PC MR slice placement that optimizes the measurement of the two ICAs may be preferable to a single PC MR slice placement that attempts to optimize both the ICAs and VAs. The ability to accurately estimate total CBF based only on carotid measurements (one or both) is perhaps not surprising based on prior work using transcranial Doppler. While losing one carotid compromised CBF accuracy, knowledge of the posterior flow adequately compensated for this loss, reflecting the strong conservation of flow balance between the anterior and posterior circulations.

We suggest that in time-limited scanning scenarios, that the following PC MR localization be used. (A1) Acquire a low resolution, highly accelerated MR angiography scout image with sagittal and coronal maximum intensity projection (MIP) images; this can typically be performed in approximately 30 s (A2) Use the sagittal MRA image to optimize the anterior-posterior pitch of the PC MR plane for the ICAs. (A3) Use the coronal image to optimize the left-right roll of the PC MR plane for the ICAs, (A4) ensure the PC MR slice is above the external carotid artery bifurcation, and (A5) below the junction of the VAs into the basilar artery. Alternatively, if there is not adequate time to acquire the MR angiography image, it is also quite possible to use the ubiquitous initial anatomical survey image; (B1) place the PC MR imaging plane at the level of the C2 cervical vertebra, and (B2) angle it to be perpendicular to the spinal cord. Either approach will intersect all the great vessels of the neck and therefore create the images suitable for our imputation models.

**FIGURE 3:**
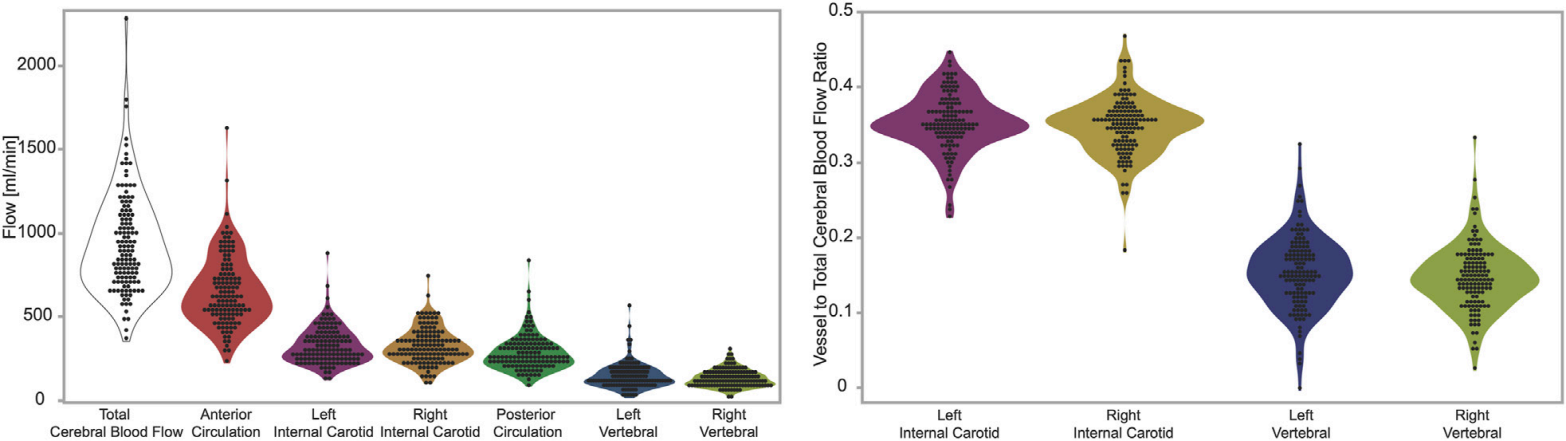
Left panel: measurements of flow in units of ml per minute. From left to right the figures shows total, anterior circulation, left internal carotid, right internal carotid, posterior circulation, left vertebral, right vertebra. Right panel: ratios of flow in individual arteries *versus* total cerebral blood flow. From left to right the figure shows left internal carotid, right internal carotid, left vertebral, right vertebra. The figures demonstrate visually (1) the range of total cerebral blood flow, (2) the contribution from individual arteries is predominantly from the anterior circulation, that flow through (3) carotid and (4) vertebral arteries are symmetric on the left and right sides.

## Limitations

Our models are only exploiting image-based information, however we know that CBF is associated with hematocrit, sex and age. (Borzage et al., 2016; Bush et al., 2016). Thus, excluding this data limits our modeling but it also allows our approach to be suitable for scenarios in which this information is either never collected, or removed to anonymize the datasets. We assumed fixed ratios between vessel flow and total CBF. This assumption is implicit on the understanding that the flow would be proportional to the volume of tissue perfused by these vessels, and that those tissues would be present in a fixed volumetric ratio. However, the ratio of these structures may change in development or senescence which limits our model application to the developing brain and provides an opportunity for further development. (Bethlehem et al., 2021). Our model assumed the PC MR slice contains the ICAs and VAs, however with an exceptionally poor localization the image might include the common carotid arteries or basilar artery. If these are incorrectly identified as ICA or VA arteries, then it would cause overestimation of the total CBF. One opportunity for future research is developing models to impute cerebral blood flow from images of common carotid or basilar arteries. Our results demonstrate flow in the two ICAs and two VAs are statistically equivalent. However, we did not include patients with systemic or cerebrovascular disease, nor patients with profound variance in the anatomy of their arteries (e.g. patients who actually lack an ICA or VA), thus our data demonstrating lateral symmetry might not generalize to populations with vascular disease. However, patients with known vascular disease or profoundly abnormal vessels may benefit from the more time-insensitive option of ASL MR. In contrast the PC MR approach is suitable for large studies and population-based screening. Moreover, our observed inter-vessel relationships could potentially be useful in recognizing deranged blood flow distribution in conditions such as steno-occlusive disease.

We did not explore other approaches to acquisition or analysis of the PC MR image data. Our secondary analysis was unable to change the prior approach to acquisition and exploring image processing approaches is beyond the scope of this project. Moreover, our imputation results are based on vascular physiology, not imaging technology. Thus, our results remain valid even with improved PC MR data acquisitions or image analyses. Our dimensionless approach means that even if our imaging methods are biased compared to approaches taken by others, they can use our imputation models. If our image acquisition methods have higher variance compared to approaches by others, our modeling error will be conservative and overestimate the variance compared to those their improved methods.

## Conclusion

Phase contrast MR is an efficient and effective way to assess total cerebral blood flow. Our results indicate that using our imputation models based on at least one ICA flow measurement provides lower variance in results than ASL, and higher intraclass correlation than test-retest PC MR. Therefore, our methods are an important set of equations describing vascular physiology. Our equations enable a new approach for dealing with real-world data and make it easier to use PC MR to obtain measurements of CBF in large numbers of patients or volunteers.

## Data Availability

The raw data supporting the conclusion of this article will be made available by the authors, without undue reservation.
